# Beneficial Impact and Molecular Mechanism of *Bacillus coagulans* on Piglets’ Intestine

**DOI:** 10.3390/ijms19072084

**Published:** 2018-07-18

**Authors:** Tao Wu, Yue Zhang, Yang Lv, Peng Li, Dan Yi, Lei Wang, Di Zhao, Hongbo Chen, Joshua Gong, Yongqing Hou

**Affiliations:** 1 Hubei Key Laboratory of Animal Nutrition and Feed Science, Wuhan Polytechnic University (WPHU), Wuhan 430023, China; wutao@whpu.edu.cn (T.W.); m13971453158@163.com (Y.Z.); yanglyu.yl@gmail.com (Y.L.); lipeng@whpu.edu.cn (P.L.); yidan810204@whpu.edu.cn (D.Y.); wanglei@whpu.edu.cn (L.W.); zhaodi@whpu.edu.cn (D.Z.); chenhongbo@whpu.edu.cn (H.C.); 2 Guelph Research and Development Centre, Agriculture and Agri-Food Canada, Guelph, ON N1G 5C9, Canada; joshua.gong@agr.gc.ca

**Keywords:** *Bacillus coagulans*, intestinal function, gut microbiota, weaned piglet

## Abstract

The aim of this research was to investigate the beneficial impact and molecular mechanism of *B. coagulans* on piglets’ intestine. Twenty-four 21 days old weaned piglets were allotted to three treatments: Control group (basal diet), B6 group (basal diet + 2 × 10^6^ CFU/g *B. coagulans*), and the B7 group (basal diet + 2 × 10^7^ CFU/g *B. coagulans*). The results showed that, compared with the control group, the B7 group had a reduced cholesterol content and gamma glutamyl transpeptidase (GGT) in plasma (*p* < 0.05); the B6 and B7 groups had a significantly decreased diarrhea rate and diamine oxidase (DAO) activity in plasma (*p* < 0.05), increased villus height in ileum and decreased crypt depth in the jejunum (*p* < 0.05); increased activities of superoxide dismutase (SOD) and catalase (CAT), and decreased the content of malondialdehyde (MDA) and H_2_O_2_ in the intestine (*p* < 0.05). These data suggested that supplementing *B. coagulans* had beneficial impacts on promoting nutrients’ metabolism, maintaining intestinal integrity, and alleviating oxidative stress and diarrhea. Further research of molecular mechanisms showed changing expression levels of related proteins and genes, suggesting that these could be involved in the regulation of the impact. The community composition of the gut microbiota was also found to be altered in several operational taxonomic units within the genus, *Prevotella* (order *Bacteroidales*), and the order, *Clostridiales.*

## 1. Introduction

In the modern intensive pig production process, piglet feeding has become one of the most important aspects [1]. Weaning of piglets involves complex events, including environmental and dietary stresses, that interfere with gut development and adaptation [2], which is one of the most critical developmental stages of the digestive tract when food is changed from maternal milk to a solid diet [3]. This is a period of starvation associated with the absence of the dam, impairment of energy status, and thermoregulation, as shown by behavioral and biochemical changes [2,3,4]. After early weaning, piglets are prone to physiological and nutritional stress reactions and the immature development of their own organs, which results in a decrease in feed intake, slow weight growth, poor mental state, and severe diarrhea [5,6]. The long-term occurrence of these symptoms leads to a high morbidity and mortality of piglets [1]. This not only causes economical losses in pig production, but also contributes to public health risks from pathogenic bacteria-infected pork products, which has been perplexing the pig breeding industry for a long time [7], especially in the post-antibiotic era.

The intestine is not only the terminal organ for digestion and absorption of dietary nutrients, but is also crucial for preventing the entry of exogenous pathogens into the systemic circulation [8]. Thus, intestinal integrity is vital to survival, growth, and health of both animals and humans. Extensive studies have demonstrated that the early-weaning of piglets can result in gut mucosal injury and dysfunction [9,10]. Neonates are prone to various stresses, particularly after early-weaning, resulting in intestinal mucosal injury and absorptive dysfunction [8,9,10,11]. The consequences of this are the occurrence of diarrhea, reduced growth, and even death, leading to a considerable economic loss [11]. Therefore, it is urgent to find and explore high quality and safe antibiotic replacement products to improve the adverse reactions caused by weaning piglet syndrome, and to promote intestinal health.

The concept of probiotics, which are considered beneficial to the gastrointestinal tract as an alternative to antibiotics, has attracted increasing interest from animal nutritionists and livestock producers [12]. It is defined as a live microbial feed supplement that is beneficial to health [13]. The beneficial effects of probiotics in recent reports is mainly reflected in these aspects: (1) Produces a large number of active enzymes in the metabolism process and promotes the absorption of nutrients, thereby, improving the conversion efficiency of feed; (2) promotes the synthesis and metabolism of proteins and vitamins; (3) inhibits the reproduction of harmful bacteria in the intestinal tract, promotes the growth of beneficial bacteria, and maintains the dynamic balance of the gut microbiota; (4) regulates the immune function of the animal body to a certain extent; and (5) reduces the irritating gas in feces, thereby, purifying the air environment and reducing pollution [14,15,16,17].

As one kind of gram positive bacteria, *Bacillus coagulans* (*B. coagulans*) is a lactic acid producing bacterial species, which is catalase positive, spore forming, motile, and a facultative anaerobe [18]. Spores of *B. coagulans* have strong resistance, resurrection, and stability, can be activated in the acidic environment of the stomach, and begin germinating and proliferating in the intestine [19,20]. Spores can adapt to the low oxygen environment in the intestinal tract and reach the gastrointestinal tract smoothly, and then can play the effect of lactic acid bacteria in the gut [20]. Because of these characteristics, *B. coagulans* is often used in veterinary applications, especially as a probiotic in cattle, poultry, and shrimp, and many studies of its beneficial effects have been continuously reported [21].

Nevertheless, there is still limited evidence suggesting whether or how *B. coagulans* could affect molecular function, promote gut health, and maintain intestinal homeostasis in piglets. Therefore, the aim of this research was to investigate the beneficial impact and molecular mechanism of *B. coagulans* on piglets’ intestines, by means of supplementing two levels of *B. coagulans* to the basal diet, and analyzing the molecular biology indexes. Moreover, this study might ultimately reveal the principle of how *B. coagulans* benefits the intestine via the maintenance of homeostasis and the regulation of biomolecular functions.

## 2. Result and Discussion

### 2.1. Effects on Growth Performance and Nutrient Metabolism

During the experimental period, the average daily gain (ADG), average daily feed intake (ADFI), and diarrhea rate were observed and calculated (Table 1). Although there was no significant difference in ADG and ADFI among the three groups, the data had a slight rise to some extent after supplementing *B. coagulans.* Moreover, the difference in diarrhea rate was remarkable. Compared with the control group, the B6 group had a significantly reduced diarrhea rate between day 0 and 10, and day 10 and 21, as well as day 0 and 21 (*p* < 0.05), and the B7 group had a significantly reduced diarrhea rate between day 0 and 10, as well as day 0 and 21 (*p* < 0.05). In addition, the diarrhea rate between day 10 and 21 in the B6 group was also exceedingly lower than that in the B7 group (*p* < 0.05).

Diarrhea is one of the most challenging problems in weaned piglet breeding. In the metabolic process, *B. coagulans* secretes the antibacterial peptide substance, coagulin, as well as lactic acid, which inhibits *Listeria*, *Micrococcus*, *Leuconostoc*, and *Enterococcus*, etc. [22]. There is evidence from animal research suggesting that *B. coagulans* is effective in both treating and preventing the recurrence of *Clostridium difficile* associated diarrhea [23]. In this study, we found the diarrhea rate was significantly reduced by the supplementation of *B. coagulans*, and 2 × 10^6^ CFU/g *B. coagulans* had a better effect in the second half of the trial. 

Plasma biochemical indicators were detected to reflect the metabolic function of piglets, with the results shown in Table 2. Compared with the control group, the B7 group had an obviously decreased total cholesterol (CHOL) and gamma glutamyl transpeptidase (GGT) and increased triglycerides (TG) in plasma (*p* < 0.05). 

Cholesterol levels in the blood are one of the routine blood testing indicators that is associated with many cardiovascular diseases [24]. Blood cholesterol is the main cause of atherosclerosis, and either high or low levels of cholesterol can lead to the damage of animal health [25]. GGT is mainly derived from the secretion of liver mitochondria, which is an essential enzyme for the metabolic process of amino acids and proteins [26]. GGT is also an indicator of oxidative stress that reflects the damage of oxygen free radicals to a variety of cells, and elevated GGT indicates damage to the liver and bile duct epithelium [27]. The results of this study showed that supplementing *B. coagulans* has the effect of lowering blood cholesterol, regulating the metabolism of amino acids and proteins, and decreasing stress reactions in the liver. Of note, although the triglycerides in plasma had a significant difference in this test, the content was still in the normal range.

In summary, the experiments of the effects on growth performance and nutrient metabolism indicated that, after weaning, supplementing *B. coagulans* could alleviate piglets’ diarrhea, regulate the metabolism of nutrients, and mildly rise ADG and ADFI. For this, the analysis of the gut microbiota and expression levels of relative proteins and genes was carried out next to reveal this regulation mechanism further.

### 2.2. Effects on Intestinal Integrity and Redox Status

**Figure 1:**
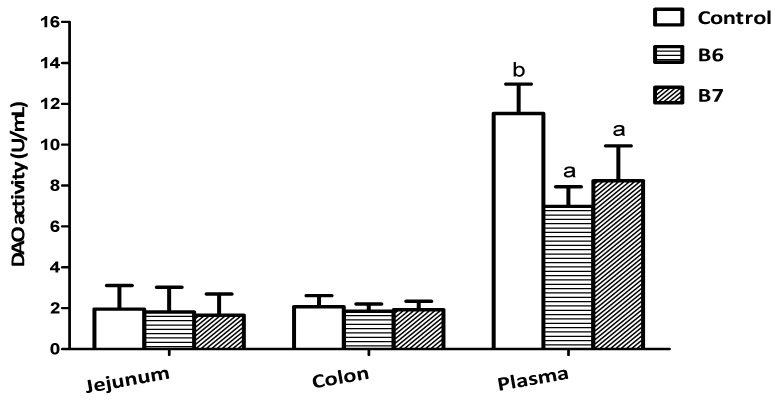
Effects of *B. coagulans* on diamine oxidase (DAO) activity in the jejunim, colon, and plasma. ^a,b^ Values within a row with different letters differ (*p* < 0.05).

Diamine oxidase (DAO) activity is frequently used as a noninvasive biomarker of alterations in the function and structure of the intestinal mucosa [28]. Under certain conditions, cells in the intestinal mucosa experience necrosis, and slough off into the enteric entocoele, resulting in a decline of DAO levels in the intestinal mucosa and an increase of DAO levels in circulation [29]. In this research (Figure 1), both the B6 and B7 groups markedly reduced the activity of DAO in plasma (*p* < 0.05), indicating that supplementing *B. coagulans* alleviated the intestinal damage caused by weaning stress.

The data of the intestinal morphology was to reflect the intestinal mucosal integrity and injury, and are summarized in Table 3. Compared with control group, both the B6 and B7 groups significantly decreased the crypt depth and increased the ratio of villus height to crypt depth in the jejunum (*p* < 0.05), and significantly increased the villus height, as well as the ratio, in the ileum (*p* < 0.05). The height of the villus and the depth of the crypt directly reflect the function of the intestinal tract. The atrophy of the villus of the small intestine leads to a decrease in the number of mature epithelial cells, resulting in the nutrients not being fully absorbed by the intestine [30]. The depth of the crypt reflects the formation rate of villus epithelial cells, and the shallow crypt indicates the rate of cell maturation is increased and the secretory function is enhanced [31]. The data of this study showed that supplementing *B. coagulans* enhanced the repair of intestinal damage caused by weaning stress, thus, preserving the integrity of the intestinal mucosa.

The intestinal redox status was determined to reflect the oxidative stress and antioxidative function of piglets supplemented with *B. coagulans* (Table 4), including the activity of superoxide dismutase (SOD) and catalase (CAT), and the content of malondialdehyde (MDA) and hydrogen peroxide (H_2_O_2_). Compared with the control group, the B6 group remarkably increased the activity of SOD in the duodenum and jejunum, as well as CAT in the colon, and decreased the content of MDA in the ileum and colon, as well as H_2_O_2_ in the jejunum and colon (*p* < 0.05); the B7 group markedly increased the activity of SOD in the jejunum, as well as CAT in the jejunum and colon, and decreased the content of MDA and H_2_O_2_ in the colon (*p* < 0.05). In addition, the difference of SOD, CAT, and H_2_O_2_ in the B6 group was larger than that in the B7 group.

Oxidative stress reflects the unbalance between the systematic phenomenon of reactive oxygen species and the capacity of the biosystem to readily detoxify the reactive intermediaries or to renovate the resulting injury that frequently occurs after piglets’ weaning [32,33], whereas, cells protect themselves from hydroxyl radicals and other oxygenants by antioxidant enzymes, including SOD and CAT [33,34]. MDA can induce noxious stress in cells and constitute homopolar protein adducts, known as advanced lipoxidation end-products (ALEs), which are usually utilized as a marker to evaluate the oxidant stress levels in a biosome [35]. H_2_O_2_ is the main product of oxidative stress in the body [36]. The results of the redox status showed that supplementing *B. coagulans* alleviated oxidative stress and enhanced the antioxidative capacity of the intestine. Furthermore, supplementing 2 × 10^6^ CFU/g *B. coagulans* had a better effect in this test.

In summary, the experiments of the effects on the intestinal integrity and redox status indicated that, after weaning, supplementing *B. coagulans* could alleviate intestinal damage and oxidative stress, maintain intestinal integrity, and enhance antioxidative capacity. For this, the expression levels of relative proteins and genes was determined next to reveal this regulation and resistant mechanism further.

### 2.3. Regulation of Protein Expression

**Figure 2:**
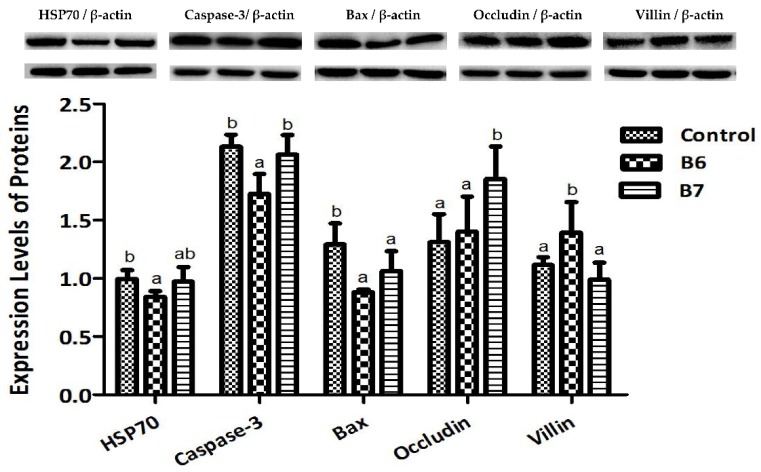
Effects of *B. coagulans* on the expression levels of proteins in the jejunum. ^a,b^ Values within a row with different letters differ (*p* < 0.05).

The expression levels of six proteins in the jejunum were tested to analyze the regulation of *B. coagulans* on the intestinal mucosal stress status and barrier function (Figure 2). Relative to the control group, the B6 group had significantly reduced protein expression levels of HSP70, Caspase-3, and Bax, and raised expression levels of Villin (*p* < 0.05); the B7 group had a significantly reduced protein expression level of Bax, and a raised expression level of Occludin (*p* < 0.05).

In response to stress, HSP70 is expressed at elevated levels to promote refolding and prevent aggregation of partially-denatured proteins, thereby, protecting cells from injury [37]. Caspase-3 is commonly activated by numerous “death” signals to cleave a variety of important cellular proteins that are responsible for the proteolytic cleavage of many key “death” proteins [38]. Bax resides in the outer mitochondrial membrane, and it promotes cell death directly through its putative function as a channel protein versus indirectly by inhibiting cellular regulators of the cell death proteases (caspases) [38,39]. In this research, we found the expression levels of these three proteins were remarkably decreased by *B. coagulans*, indicating that *B. coagulans* had a beneficial effect on regulating the related proteins to protect the intestine from stresses and injury, which is one of the mechanisms of resisting and alleviating weaning and oxidative stress.

Villin is one kind of actin binding protein and a marker of villus cell differentiation, which is conducive to prop up the microfilaments of the microvilli of the mucosal villus [40]. Occludin integrates such diverse processes as gene transcription, tumor suppression, and cell proliferation to modulate the intestinal mucosal structure and function [41]. After supplementing *B. coagulans*, the expression of these two proteins were regulated and obviously increased, indicating that *B. coagulans* had a beneficial effect on maintaining the intestinal barrier function and promoting the growth of villus, which might be exactly one of the key mechanisms of alleviating intestinal injury and diarrhea.

### 2.4. Regulation of Gene Expression

The expression levels of genes associated with intestinal immunity, inflammation, transportation, and absorption were tested to analyze the regulation of *B. coagulans* on the relative function (Table 5). Compared with the control group, the B6 group obviously increased the expression levels of *IFN-α*, *IFN-γ*, *OAS1*, *MX2*, *IL-4*, *CCL-2*, *AQP3*, and *LPL* in the ileum, and *IFN-β*, *SGLT-1* and *b^0,+^AT* in the colon, while decreasing the expression levels of *IL-4*, *CCL-2*, and *IFN-γ* in the colon (*p* < 0.05); the B7 group remarkably increased the expression levels of *IFN-α*, *IFN-β*, *OAS1*, *MX2*, and *AQP3* in the ileum and *MX1*, *AQP3*, *SGLT-1*, *LPL*, *INSR*, and *b^0,+^AT* in the colon, while decreasing the expression levels of *CXCL-9* in the ileum and *CXCL-9*, *IFN-γ*, and *IL-1β* in the colon (*p* < 0.05).

The cellular response to viral infection includes the induction of genes for the type I interferons, *IFN-α* and *IFN-β*, which are produced in most cell types and play a vital role in innate resistance to viral and bacterial infections [42]. *IFN-α/β* can induce the expression of genes encoding for antiviral proteins, particularly myxovirus (MX) and 2′-5′ oligoadenylate synthetases (OAS) [43,44]. *IFNγ*, or type II interferon, is a cytokine that is critical for innate and adaptive immunity against viral, some bacterial, and protozoal infections [45]. In this research, we found there were marked alterations to the expression levels of these genes after supplementing *B. coagulans*, and the results indicated that *B. coagulans* regulated immune-related genes to improve immunity and inhibit pathogens in the intestine, which is one of the main mechanisms of lowering the diarrhea rate.

Oxidative stress is associated with early weaning, and it is suggested that oxidative stress may be one of the main causes of early weaning syndrome [46]. Oxidative stress has been implicated in the development of many chronic inflammatory disorders, such as enteritis, myocarditis, and thyroiditis [47]. Antioxidant defense systems may be impaired as a consequence of excessive oxidative stress, and inflammatory responses can be partially mediated by oxidative stress [48]. In this study, some inflammatory cytokines were remarkably changed after supplementing *B. coagulans*, including *IFN-γ*, *IL-1β*, *IL-4*, *CXCL-9*, and *CCL-2*, implying that the beneficial impact of *B. coagulans* on alleviating weaning and oxidative stress was regulated by altering the expression levels of these inflammatory cytokines. 

*LPL* (lipoprotein lipase) is expressed in heart, muscle, and adipose tissue, and it acts as a homodimer, obtaining the double functions of triglyceride hydrolase and ligand/bridging factor for receptor-mediated lipoprotein uptake [49]. *INSR* (insulin receptor) is a transmembrane receptor activated by insulin, including IGF-I and IGF-II. The binding of insulin or other ligands to this receptor activates the insulin signaling pathway, which regulates glucose uptake and release, as well as the synthesis and storage of carbohydrates, lipids, and proteins [50]. *AQP3* is a selective aquaporin and is mainly distributed in intestinal epithelial cells, which can rapidly absorb water in the intestinal cavity into the blood and alter the endocrine environment of the intestinal cavity [51]. *SGLT-1* (sodium glucose cotransporters-1) is a high affinity/low capacity transporter of glucose in the mammalian small intestine and kidneys, and is responsible for the entire glucose absorption in the small intestine [52]. *b^0,+^AT* (*b^0,+^* amino acid transporter) plays a role in the high-affinity and sodium-independent transport of cystine and neutral and dibasic amino acids, and appears to function in the reabsorption of cystine in the kidney tubule [53]. After supplementing *B. coagulans*, the expression of genes related to nutrients’ absorption and transportation were significantly changed, implying that the beneficial impact of *B. coagulans* on promoting nutrients’ metabolism was regulated by altering the expression levels of the identified genes. 

### 2.5. Regulation of Gut Microbiota

**Figure 3:**
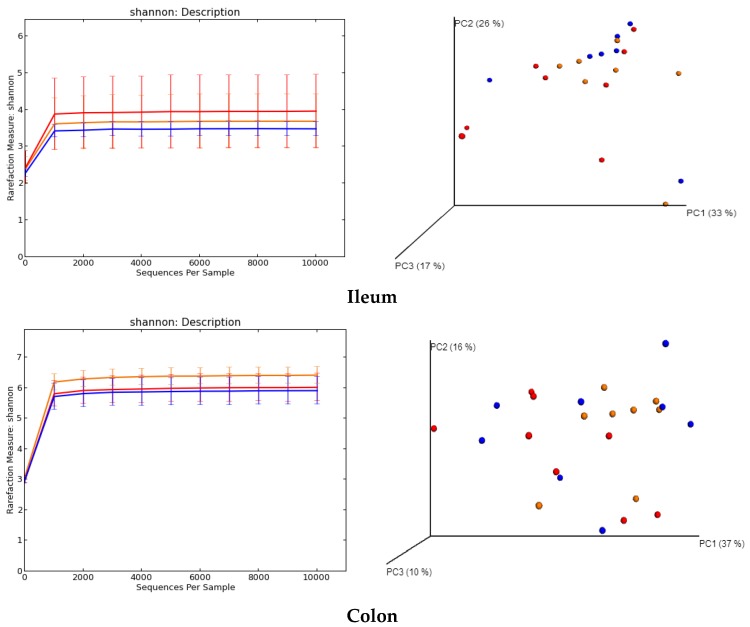

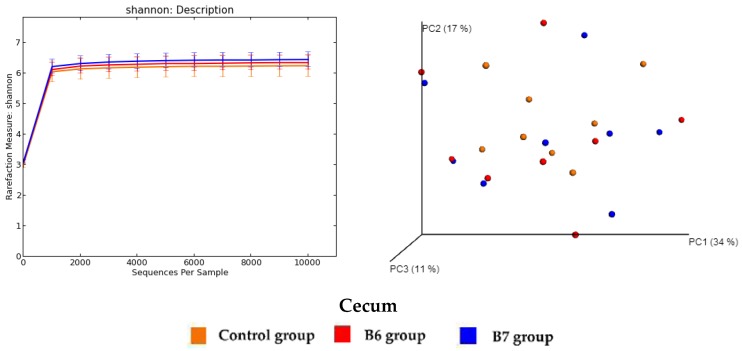
The Shannon α-diversity index (rarefaction curves) and β-diversity (weighted UniFrac principal component analysis).

The diversity analysis of the gut microbiota in the ileum, colon, and cecum are shown in Figure 3. There was a significant difference in the Shannon α-diversity index between the control group (6.43 ± 0.27) and the B7 group (5.92 ± 0.45) in the colon (*p* = 0.039), but no difference among the three groups in the ileum and cecum. There was a significant difference in the β-diversity (weighted Unifrac) between the control and the B6 group (*p* = 0.021), as well as between the B6 and B7 group (*p* = 0.021), in the ileum, between the control and the B7 group in the colon (*p* = 0.015), and between the control and the B6 group (*p* = 0.036), as well as between the control and the B7 group (*p* = 0.001) in the cecum.

A total of 1,313,016 reads were obtained from the ileum in the three groups, with 407,289 reads from the control group, 558,738 from the B6 group, and 346,989 from the B7 group. A total of 2,003,105 reads were obtained from the colon, 707,573 reads from the control group, 650,742 from the B6 group, and 651,835 from the B7 group. A total of 3,521,123 reads were obtained from the cecum, 902,813 reads from the control group, 1,213,060 from the B6 group, and 1,405,250 from the B7 group. The relative abundance of the OTUs (Operational Taxonomic Units) is summarized in Table 6. There were four OTUs with a significant difference in the ileum, 17 significant OTUs in the colon, and 15 significant OTUs in the cecum.

**Figure 4:**
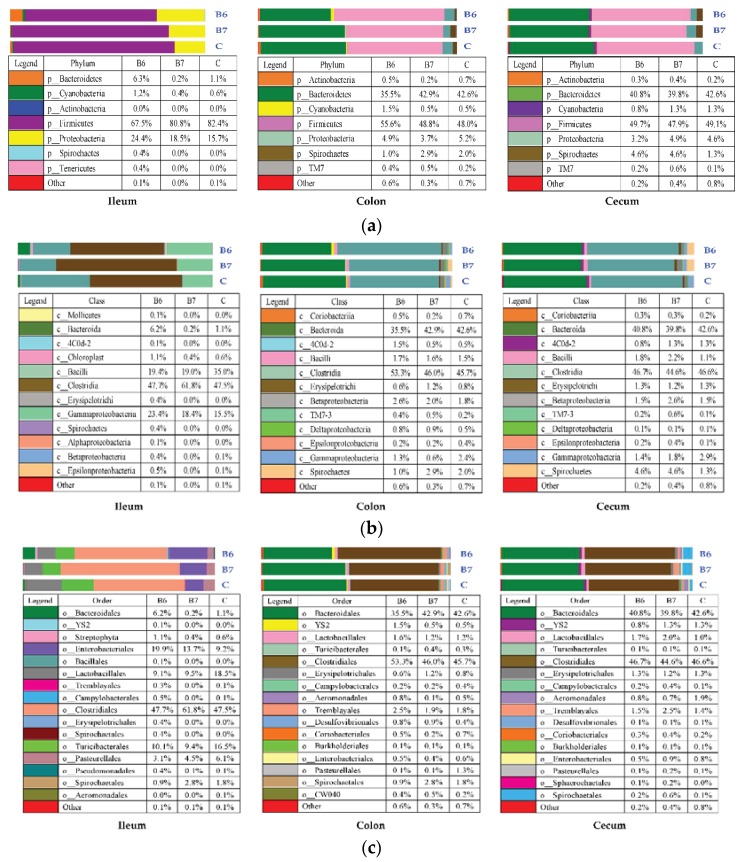

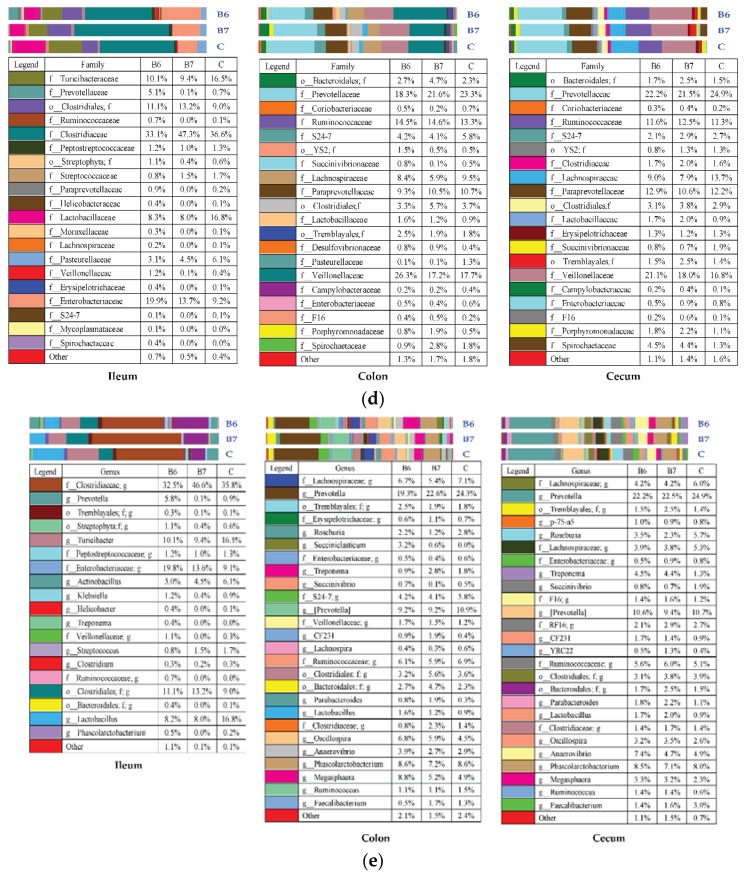
(**a**) The community composition at the phylum level; (**b**) the community composition at the class level; (**c**) the community composition at the order level; (**d**) the community composition at the family level; and (**e**) the community composition at the genus level.

The mean relative abundances of the different predominant taxa at five levels in the community composition of each group are shown in Figure 4a–e. The dominant bacteria at the phylum level were *Firmicutes* and *Proteobacteria* in the ileum, and *Bacteroidetes* and *Firmicutes* in the colon and cecum. The dominant bacteria at the class level were *Bacilli*, *Clostridia*, and *Gammaproteobacteria* in the ileum, and *Bacteroidia* and *Clostridia* in the colon and cecum. The dominant bacteria at the order level were *Bacteroidales*, *Enterobacteriales*, *Clostridiales*, *Lactobacillales*, and *Turicibacterales* in the ileum, and *Bacteroidales* and *Clostridiales* in the colon and cecum. The dominant bacteria at the family level were *Turicibacteraceae*, *Lactobacillaceae*, o_*Clostridiales*. f (an unassigned family that belongs to the order *Clostridiales*), *Clostridiaceae,* and *Enterobacteriaceae* in the ileum, and *Prevotellaceae*, *Ruminococcaceae*, *Veillonellaceae*, *Paraprevotellaceae*, and *Lachnospiraceae* in the colon and cecum. The dominant bacteria at the genus level were *Turicibacter*, *Clostridiales.* f. g, *Clostridiaceae.* g, and *Enterobacteriaceae.* g in the ileum, and *Lactobacillus* and *Prevotella* in the colon and cecum.

In the gut microbiota of mammals, *Firmicutes* and *Bacteroidetes* were the dominant phyla, followed by *Fusobacteria*, *Proteobacteria*, and *Actinobacteria* [54]. Previous studies have also presented the similar result that *Firmicutes* and *Bacteroidetes* were still the main phyla in pigs regardless of the growing ages or different intestinal segments [55]. Despite the variations showed in Figure 4a–e, only 36 OTUs underwent statistically significant changes among the groups (Table 6) and most of them belong to the genus, *Prevotella,* or are included in the order, *Clostridiales*, specifically in three of its families (*Clostridiaceae*, *Lachnospiraceae*, and *Ruminocccaceae*) and a very close one (*Veillonellaceae*). *Prevotella* tend to colonize animals and the human gut, and may cause infections, but can also co-exist harmlessly with their human host [56]. It is increasingly gaining attention as a commensal microbe in the intestine because of its ability to degrade a broad spectrum of plant polysaccharides [57]. The *Veillonellaceae* family are mainly bacteria related to the metabolism of nutrients, especially the metabolism of amino acids [58]. The *Lachnospiraceae* and *Ruminococcaceae* families are common gut microbes that break down complex carbohydrates, and they are most common in the digestive tracts of animals with carb-heavy diets. This is usually good for ruminants, which have a great difference to pigs [59,60]. In this study, the composition of some OTUs belonging to *Prevotella, Veillonellaceae*, *Lachnospiraceae,* and *Ruminococcaceae* had significant differences, indicating that supplementing *B. coagulans* could benefit and optimize the digestion and absorption of nutrients, thus, reducing anyt unnecessary waste of nutrients. The family, *Clostridiaceae,* belongs to the phylum, *Firmicutes,* and includes 40 genera. Members belonging to this family were isolated mainly from the environment and from commensal digestive microbiota of mammals. Many of them, particularly *Clostridium,* are the major pathogens of people and animals [61]. After supplementing *B. coagulans*, the composition of the genus, *Clostridium,* had a decrease in the colon, indicating that *B. coagulans* might have the capacity to inhibit the growth and reproduction of pathogenic microorganisms.

## 3. Material and Methods

### 3.1. Experimental Design and Sample Collection

The animal-use protocol for this research was approved by the Animal Care and Use Committee of the Hubei Province. The 24 healthy crossbred piglets (Duroc × Landrace × Yorkshire) were weaned at 21 day of age and fed with a corn and soybean meal-based diet. Each piglet was individually housed in a 1.20 × 1.10 m^2^ steel metabolic cage. After a period of 3 days adaptation, piglets were assigned randomly on the basis of body weight and litter origin to 3 groups: (1) The control (C) group in which piglets were fed with the basal diet; (2) the B6 group in which piglets were fed with the basal diet supplemented with 2.0 × 10^6^ CFU/g *B. coagulans*; and (3) the B7 group in which piglets were fed with the basal diet supplemented with 2.0 × 10^7^ CFU/g *B. coagulans*. All diets were isocaloric [62]. On the 21^st^ day of the trial, blood, intestine, and digesta samples were collected and stored at −80 °C until assay according to a previous study [63].

*B. coagulans* were produced by fermenting; the conditions were 200 r/min of stirring speed and 350 L/h of throughput. The products of fermentation were filtered by organic ceramic membrane filtration equipment, and mixed with mineral adsorbent by 1:1. Final products were a dry powder that were counted by the plate counting method to determine the dosage and then were mixed in the diet.

### 3.2. Plasma Biochemical Indicators and Intestinal Redox Status

Plasma biochemical indicators were measured with corresponding kits using a Hi-tachi 7060 Automatic Biochemical Analyzer (Hitachi, Tokyo, Japan), and the activities of DAO, SOD, and CAT, and the content of MDA and H_2_O_2_ were determined using commercially available kits (Jiancheng Bioengineering Institute, Nanjing, China). Assays were performed in triplicate. 

### 3.3. Intestinal Morphology

To determine the intestinal morphology, paraformaldehyde-fixed jejunum and ileum samples were dehydrated and embedded in paraffin. 5-μm sections were cut and then stained with hematoxylin and eosin stain. Intestinal morphology was determined using a light microscope (Leica, Solms, Germany), with the Leica Application Suite image analysis software (Leica, Solms, Germany). The villus area was quantitated from the perimeter and height of the villi. The ratio of the villus height to crypt depth was calculated. 

### 3.4. Expression Levels of Proteins

The expression levels of proteins were performed using western blotting as described by Hou et al. [64]. The primary antibodies: HSP70, Caspase-3, Bax, Villin, and Occludin (rabbit, 1:1000; Cell Signaling Technology, Inc., Danvers, MA, USA), β-actin (mouse 1:2000; Sigma-Aldrich Inc., St. Louis, MI, USA). The secondary antibody: Anti-rabbit (mouse, 1:2000; Zhongshan Golden Bridge Biological Technology Co., Ltd., Beijing, China). Blots were carried out by utilizing a chemiluminescence kit (Amersham Biosciences, Uppsala, Sweden) and image forming system (Alpha Innotech, New York, NY, USA).

### 3.5. Expression Levels of Genes

The gene expression levels were quantitated by the method of real-time PCR as described by Yi et al. [65]. The real-time PCR was carried out with primers (Table 7) of these genes, as well as the reference gene, ribosomal protein L 4 (RPL4), and the SYBR^®^ Premix Ex Taq™ (Takara, Dalian, China) on 7500 Fast Real-Time PCR System (Applied Biosystems, Foster City, CA, USA). Data was analyzed by the 2^−ΔCt^ method as described by [65].

### 3.6. Analysis of Gut Microbiota 

Total bacterial DNA was extracted, the gene-specific sequences targeted the 16S V3 and V4 regions and were amplified with two stage PCR, and then were analyzed by MiSeq sequencing. The results were processed with QIIME as described by Caporaso et al. [66].

α-Diversity metrics were calculated using a read depth of 10,000 and a β-diversity distance matrix was calculated based on thr UniFrac metric, which was used for the principal coordinates analysis [67]. The significance of the diet effect on the β-diversity distance matrix was assessed by PERMANOVA analysis [67]. Raw sequence data and detection and removal of chimeras were performed using the software, USEARCH and UCHIIME [66,68]. 

### 3.7. Statistical Analysis

Data were analyzed using a one-way analysis of variance to analysis, expressed as mean values ± SEM. All experimental data was analysed using SPSS (Version 17.0, SPSS Inc., Chicago, IL, USA). A *p*-value of <0.05 was considered statistically significant. The data of the gut microbiota were processed by QIIME platform.

## 4. Conclusions

Supplementing *B. coagulans* in the small intestine of weaning piglets had beneficial impacts on improving the growth performance, lowering the diarrhea rate, promoting nutrients’ metabolism, maintaining intestinal integrity, and alleviating oxidative stress. Further research of molecular mechanisms showed that these beneficial impacts might be regulated by changing expression levels of related proteins and genes and altering the community composition of the gut microbiota.

## Figures and Tables

**Table 1:** Effects of *B. coagulans* on growth performance of weaned piglets.

Item	Control	B6	B7
**Day 0–10**			
ADG/g	295.35 ± 70.08	334.07 ± 51.97	344.68 ± 65.62
ADFI/g	239.40 ± 84.79	263.20 ± 62.74	267.60 ± 77.96
F/G	1.28 ± 0.21	1.28 ± 0.12	1.29 ± 0.04
Diarrhea rate/%	27.0 ^b^	9.0 ^a^	5.0 ^a^
**Day 10–21**			
ADG/g	592.02 ± 122.77	642.62 ± 99.67	635.90 ± 96.64
ADFI/g	403.27 ± 119.01	432.27 ± 119.55	409.82 ± 166.78
F/G	1.48 ± 0.06	1.53 ± 0.22	1.56 ± 0.11
Diarrhea rate/%	11.8 ^b^	0.9 ^a^	5.5 ^b^
**Day 0–21**			
ADG/g	450.72 ± 96.11	497.22 ± 77.65	495.68 ± 72.39
ADFI/g	325.24 ± 97.15	342.10 ± 81.62	354.95 ± 81.05
F/G	1.40 ± 0.10	1.46 ± 0.06	1.41 ± 0.09
Diarrhea rate/%	19.5 ^b^	5.2 ^a^	5.7 ^a^

^a,b^ Values within a row with different letters differ (*p* < 0.05).

**Table 2:** Effects of *B. coagulans* on plasma biochemical indicators of weaned piglets.

Item	Control	B6	B7
TP (g/L)	49.68 ± 2.70	48.86 ± 4.17	48.16 ± 3.31
CHOL (mmol/L)	1.77 ± 0.29 ^b^	1.71 ± 0.19 ^ab^	1.51 ± 0.23 ^a^
TG (mmol/L)	0.37 ± 0.02 ^a^	0.41 ± 0.09 ^ab^	0.46 ± 0.08 ^b^
GLU (mmol/L)	5.54 ± 0.84	5.48 ± 0.16	5.51 ± 0.85
GGT (mmol/L)	36.23 ± 8.94 ^b^	32.48 ± 4.08 ^ab^	28.67 ± 5.25 ^a^

^a,b^ Values within a row with different letters differ (*p* < 0.05).

**Table 3:** Effects of *B. coagulans* on the intestinal morphology of weaned piglets.

Item	Jejunum			Ileum		
	Control	B6	B7	Control	B6	B7
villus height (μm)	318.7 ± 35.0	350.1 ± 41.4	336.6 ± 41.2	242.1 ± 22.8 ^a^	273.3 ± 19.8 ^b^	285.2 ± 30.7 ^b^
crypt depth (μm)	213.6 ± 18.2 ^b^	168.7 ± 15.8 ^a^	175.4 ± 20.2 ^a^	161.0 ± 11.1	168.0 ± 13.3	173.2 ± 19.5
villus height/crypt depth	1.49 ± 0.11 ^a^	2.08 ± 0.17^c^	1.92 ± 0.15 ^b^	1.51 ± 0.09 ^a^	1.63 ± 0.12 ^b^	1.65 ± 0.08 ^b^
villous surface area (cm^2^)	29677 ± 3031	31738 ± 3633	29,540 ± 4078	27,520 ± 932	28,396 ± 3715	28,502 ± 2870

^a,b^ Values within a row with different letters differ (*p* < 0.05).

**Table 4:** Effects of *B. coagulans* on the intestinal redox status of weaned piglets.

Item	Control	B6	B7	Control	B6	B7
	**Duodenum**			**Ileum**		
SOD (U/mg)	42.25 ± 3.76 ^a^	54.89 ± 4.19 ^b^	45.48 ± 3.30 ^a^	83.04 ± 5.79	78.95 ± 3.00	79.68 ± 4.52
CAT (U/mg)	17.92 ± 4.41	16.61 ± 2.73	15.59 ± 3.64	9.54 ± 3.08	7.93 ± 1.72	7.92 ± 1.40
MDA (nmol/mg)	4.52 ± 0.77	5.82 ± 1.77	5.69 ± 0.93	6.00 ± 2.40 ^b^	3.52 ± 1.11 ^a^	4.21 ± 1.40 ^ab^
H_2_O_2_ (nmol/mg)	5.06 ± 1.10 ^ab^	4.12 ± 1.01^a^	5.35 ± 1.21 ^b^	8.34 ± 1.80	7.77 ± 3.20	7.49 ± 1.82
	**Jejunum**			**Colon**		
SOD (U/mg)	81.55 ± 10.51 ^a^	90.53 ± 5.43 ^b^	94.21 ± 6.36 ^b^	100.54 ± 25.07	95.62 ± 4.92	92.56 ± 12.95
CAT (U/mg)	7.64 ± 1.23 ^a^	7.55 ± 1.11 ^a^	11.00 ± 2.48 ^b^	6.56 ± 1.15 ^a^	10.58 ± 2.40 ^b^	11.79 ± 2.89 ^b^
MDA (nmol/mg)	11.71 ± 4.75	9.61 ± 4.82	13.39 ± 3.96	3.66 ± 1.74 ^b^	1.64 ± 0.45 ^a^	2.30 ± 0.69 ^a^
H_2_O_2_ (nmol/mg)	30.75 ± 10.31 ^b^	19.40 ± 5.24 ^a^	23.08 ± 5.72 ^ab^	25.25 ± 6.43 ^b^	19.61 ± 5.00 ^a^	16.80 ± 3.04 ^a^

^a,b^ Values within a row with different letters differ (*p* < 0.05).

**Table 5:** Regulation of *B. coagulans* on the gene expression in the ileum and colon.

Item	Ileum			Colon		
	Control	B6	B7	Control	B6	B7
*IFN-α*	1.000 ± 0.156 ^a^	1.393 ± 0.211 ^b^	2.124 ± 0.383 ^c^	1.000 ± 0.218	1.041 ± 0.220	1.016 ± 0.323
*IFN-β*	1.000 ± 0.239 ^a^	1.247 ± 0.219 ^ab^	1.337 ± 0.313 ^b^	1.000 ± 0.141 ^a^	2.436 ± 0.483 ^b^	0.905 ± 0.205 ^a^
*IFN-γ*	1.000 ± 0.167 ^a^	1.276 ± 0.202 ^b^	1.191 ± 0.302 ^ab^	1.000 ± 0.223 ^b^	0.825 ± 0.159 ^a^	0.682 ± 0.078 ^a^
*MX1*	1.000 ± 0.135	1.000 ± 0.230	1.196 ± 0.271	1.000 ± 0.172 ^a^	0.937 ± 0.159 ^a^	1.194 ± 0.188 ^b^
*MX2*	1.000 ± 0.144 ^a^	2.015 ± 0.264 ^b^	2.649 ± 0.482 ^c^	1.000 ± 0.239	0.806 ± 0.203	0.976 ± 0.236
*OAS1*	1.000 ± 0.148 ^a^	1.437 ± 0.313 ^b^	1.467 ± 0.354 ^b^	1.000 ± 0.130	1.088 ± 0.212	1.001 ± 0.263
*IL-1β*	1.000 ± 0.214	1.022 ± 0.125	0.846 ± 0.185	1.000 ± 0.257 ^b^	0.984 ± 0.113 ^b^	0.708 ± 0.130 ^a^
*IL-4*	1.000 ± 0.265 ^a^	1.540 ± 0.300 ^b^	1.291 ± 0.285 ^ab^	1.000 ± 0.168 ^b^	0.759 ± 0.166 ^a^	0.870 ± 0.229 ^ab^
*CXCL-9*	1.000 ± 0.253 ^b^	0.868 ± 0.119 ^ab^	0.787 ± 0.158 ^a^	1.000 ± 0.204 ^b^	1.102 ± 0.269 ^b^	0.729 ± 0.186 ^a^
*CCL-2*	1.000 ± 0.205 ^a^	1.360 ± 0.325 ^b^	1.143 ± 0.275 ^ab^	1.000 ± 0.250 ^b^	0.646 ± 0.096 ^a^	0.862 ± 0.168 ^b^
*AQP3*	1.000 ± 0.217 ^a^	2.643 ± 0.708 ^b^	2.382 ± 0.602 ^b^	1.000 ± 0.233 ^a^	0.923 ± 0.230 ^a^	1.287 ± 0.265 ^b^
*SGLT-1*	1.000 ± 0.232 ^ab^	0.843 ± 0.132 ^a^	1.199 ± 0.268 ^b^	1.000 ± 0.203 ^a^	1.340 ± 0.273 ^b^	1.358 ± 0.223 ^b^
*LPL*	1.000 ± 0.203 ^a^	1.307 ± 0.276 ^b^	1.156 ± 0.216 ^ab^	1.000 ± 0.195 ^a^	0.857 ± 0.127 ^a^	1.250 ± 0.333 ^b^
*INSR*	1.000 ± 0.244	1.104 ± 0.275	1.037 ± 0.206	1.000 ± 0.203 ^a^	1.016 ± 0.186 ^a^	1.390 ± 0.311 ^b^
*b^0,+^AT*	1.000 ± 0.257	1.017 ± 0.219	1.228 ± 0.211	1.000 ± 0.215 ^a^	1.678 ± 0.390 ^b^	1.521 ± 0.370 ^b^

^a,b,c^ Values within a row with different letters differ (*p* < 0.05).

**Table 6:** Relative abundance of the operational taxonomic units (OTUs).

OTU	*p* Value	FDR *p* Value	Relative Abundance			Taxonomy
			C	B6	B7	
**Ileum**						
4406684	0.003	0.845	6.000	5.125	0.143	o_*Bacteroidales*; f_*Prevotellaceae*; g_*Prevotella*
299382	0.019	0.877	0.143	46.875	0.000	o_*Bacteroidales*; f_*Prevotellaceae*; g_*Prevotella*
OTU8146	0.020	0.877	0.714	0.000	1.857	o_*Clostridiales*; f_*Clostridiaceae*; g_
289257	0.038	0.877	2.857	13.875	1.571	o_*Bacteroidales*; f_*Prevotellaceae*; g_*Prevotella*;
**Colon**						
355450	0.012	0.943	22.250	14.000	2.000	o_*Clostridiales*; f_*Ruminococcaceae*; g_
OTU128	0.013	0.943	0.000	48.500	4.250	o_*Bacteroidales*; f_*Prevotellaceae*; g_*Prevotella*
613933	0.015	0.943	3.500	7.375	1.375	o_*Clostridiales*; f_; g_
187350	0.017	0.943	2.375	0.125	3.125	o_*Clostridiales*; f_; g_
325236	0.021	0.943	0.375	2.625	5.875	o_*Clostridiales*; f_; g_
300859	0.022	0.943	61.125	31.625	30.875	o_*Bacteroidales*; f_*Prevotellaceae*; g_*Prevotella*
36705	0.025	0.943	2.625	1.625	1.250	o_*Clostridiales*; f_*Clostridiaceae*; g_*Clostridium*
4392188	0.026	0.943	7.375	1.375	1.875	o_*Clostridiales*; f_*Lachnospiraceae*; g_
OTU16964	0.031	0.943	0.500	2.875	0.000	o_*Clostridiales*; f_*Veillonellaceae*; g_*Acidaminococcus*
301253	0.032	0.943	2.250	1.250	0.000	o_*Bacteroidales*; f_*Prevotellaceae*; g_*Prevotella*
174019	0.033	0.943	1.125	10.625	1.250	o_*Clostridiales*; f_*Lachnospiraceae*; g_*Coprococcus*
4481427	0.034	0.943	7.625	0.125	0.000	o_*Clostridiales*; f_*Lachnospiraceae*; g_*Roseburia*
151623	0.038	0.943	6.250	42.250	12.875	o_*Clostridiales*; f_*Veillonellaceae*; g_*Megasphaera*
4416951	0.038	0.943	0.375	9.500	4.500	o_*Clostridiales*; f_*Ruminococcaceae*; g_*Ruminococcus*
4295618	0.041	0.943	7.750	3.000	0.750	o_*Bacteroidales*; f_*Prevotellaceae*; g_*Prevotella*
295835	0.047	0.943	7.625	19.500	0.125	o_*Clostridiales*; f_; g_
OTU1089	0.049	0.943	0.250	1.375	3.500	o_*Bacteroidales*; f_*Prevotellaceae*; g_*Prevotella*
**Cecum**						
OTU100	0.012	0.999	0.500	10.625	8.250	o_*Bacteroidales*; f_*Prevotellaceae*; g_*Prevotella*
OTU102	0.013	0.999	0.250	14.625	9.875	o_*Bacteroidales*; f_*S24-7*; g_
174153	0.016	0.999	0.375	3.750	4.000	o_*Clostridiales*; f_*Lachnospiraceae*; g_
196800	0.019	0.999	0.250	6.625	5.250	o_*Bacteroidales*; f_*Prevotellaceae*; g_*Prevotellastercorea*
3275562	0.020	0.999	79.625	24.000	25.375	o_*Clostridiales*; f_*Lachnospiraceae*; g_
190320	0.028	0.999	71.500	19.500	22.250	o_*Clostridiales*; f_*Lachnospiraceae*; g_*Roseburiafaecis*
4301511	0.034	0.999	3.625	0.125	0.000	o_*Clostridiales*; f_; g_
310301	0.037	0.999	0.000	8.625	15.000	o_*Clostridiales*; f_*Ruminococcaceae*; g_*Faecalibacteriumprausnitzii*
183030	0.038	0.999	6.500	0.000	0.000	o_*Clostridiales*; f_; g_
4416951	0.038	0.999	0.000	3.375	3.750	o_*Clostridiales*; f_*Ruminococcaceae*; g_*Ruminococcus*
4326866	0.039	0.999	0.750	4.250	3.000	o_*Clostridiales*; f_*Ruminococcaceae*; g_
OTU34	0.039	0.999	0.000	50.750	38.500	o_*Bacteroidales*; f_*Prevotellaceae*; g_*Prevotella*
4392188	0.046	0.999	17.000	3.500	2.750	o_*Clostridiales*; f_*Lachnospiraceae*; g_
1110312	0.047	0.999	11.250	2.250	3.125	o_*Clostridiales*; f_; g_
22697	0.049	0.999	0.500	82.125	82.625	o_*Clostridiales*; f_*Veillonellaceae*; g_*Succiniclasticum*

**Table 7:** Primers for real-time PCR analysis.

Gene	Forward	Reverse
*RPL4*	GAGAAACCGTCGCCGAAT	GCCCACCAGGAGCAAGTT
*IFN-α*	ACTCCATCCTGGCTGTGAGGAAAT	ATCTCATGACTTCTGCCCTGACGA
*IFN-β*	ATGTCAGAAGCTCCTGGGACAGTT	AGGTCATCCATCTGCCCATCAAGT
*IFN-γ*	TCTGGGAAACTGAATGACTTCG	GACTTCTCTTCCGCTTTCTTAGGTT
*MX1*	AGTGCGGCTGTTTACCAAG	TTCACAAACCCTGGCAACTC
*MX2*	CGCATTCTTTCACTCGCATC	CCTCAACCCACCAACTCACA
*OAS1*	TGGTGGTGGAGACACACACA	CCAACCAGAGACCCATCCA
*IL-1β*	CAACGTGCAGTCTATGGAGT	GAGGTGCTGATGTACCAGTTG
*IL-4*	AGGAGCCACACGTGCTTGA	TTGCCAAGCTGTTGAGATTCC
*CXCL-9*	CTTGCTTTTGGGTATCATCTTCCT	TCATCCTTTGGCTGGTGTTG
*CCL-2*	CATAAGCCACCTGGACAAGAAAA	GGGTATTTAGGGCAAGTTAGAAGGA
*AQP3*	AAGCTGTCCCAAGTAAAGCACAA	GCCCTACTTCCTGTTTCACCAC
*SGLT-1*	CCCAAATCAGAGCATTCCATTCA	AAGTATGGTGTGGTGGCCGGTT
*LPL*	AGCCTGAGTTGGACCCATGT	CTCTGTTTTCCCTTCCTCTCTCC
*INSR*	GGGGCTAAAGAGGAACTATGAGG	AGAGGAAAGCGAAGACAGGAAA
*b^0,+^AT*	CGAGTACCCGTACCTGATGGA	TGCGTAGAAGGGCGAAGAA
